# Effects of Laccase and Transglutaminase on the Physicochemical and Functional Properties of Hybrid Lupin and Whey Protein Powder

**DOI:** 10.3390/foods13132090

**Published:** 2024-07-01

**Authors:** Teguh Santoso, Thao M. Ho, Geerththana Vinothsankar, Kirsi Jouppila, Tony Chen, Adrian Owens, Masoumeh Pourseyed Lazarjani, Mustafa M. Farouk, Michelle L. Colgrave, Don Otter, Rothman Kam, Thao T. Le

**Affiliations:** 1 AUT Centre for Future Foods, Auckland University of Technology, Auckland 1010, New Zealand; 2 School of Science, Auckland University of Technology, Auckland 1010, New Zealand; 3 Department of Food and Nutrition, University of Helsinki, P.O. Box 66, 00014 Helsinki, Finland; 4 Helsinki Institute of Sustainability Science (HELSUS), University of Helsinki, P.O. Box 65, 00014 Helsinki, Finland; 5 Food Technology and Processing, Smart Foods & Bioproducts, AgResearch Ltd., Grasslands Research Centre, Palmerston North 4440, New Zealand; 6 CSIRO Agriculture and Food, 306 Carmody Rd., St. Lucia, QLD 4067, Australia; 7 Australian Research Council Centre of Excellence for Innovations in Peptide and Protein Science, School of Science, Edith Cowan University, Joondalup, WA 6027, Australia; 8 DEO Dairy Consulting, Marton 4787, New Zealand

**Keywords:** hybird protein, lupin flour, whey protein concentrate, enzymatic cross-linking, laccase, transglutaminase, physicochemical properties, functional properties

## Abstract

Plant-based protein is considered a sustainable protein source and has increased in demand recently. However, products containing plant-based proteins require further modification to achieve the desired functionalities akin to those present in animal protein products. This study aimed to investigate the effects of enzymes as cross-linking reagents on the physicochemical and functional properties of hybrid plant- and animal-based proteins in which lupin and whey proteins were chosen as representatives, respectively. They were hybridised through enzymatic cross-linking using two laccases (laccase R, derived from *Rhus vernicifera* and laccase T, derived from *Trametes versicolor*) and transglutaminase (TG). The cross-linking experiments were conducted by mixing aqueous solutions of lupin flour and whey protein concentrate powder in a ratio of 1:1 of protein content under the conditions of pH 7, 40 °C for 20 h and in the presence of laccase T, laccase R, or TG. The cross-linked mixtures were freeze-dried, and the powders obtained were assessed for their cross-linking pattern, colour, charge distribution (ζ-potential), particle size, thermal stability, morphology, solubility, foaming and emulsifying properties, and total amino acid content. The findings showed that cross-linking with laccase R significantly improved the protein solubility, emulsion stability and foaming ability of the mixture, whereas these functionalities were lower in the TG-treated mixture due to extensive cross-linking. Furthermore, the mixture treated with laccase T turned brownish in colour and showed a decrease in total amino acid content which could be due to the enzyme’s oxidative cross-linking mechanism. Also, the occurrence of cross-linking in the lupin and whey mixture was indicated by changes in other investigated parameters such as particle size, ζ-potential, etc., as compared to the control samples. The obtained results suggested that enzymatic cross-linking, depending on the type of enzyme used, could impact the physicochemical and functional properties of hybrid plant- and animal-based proteins, potentially influencing their applications in food.

## 1. Introduction

Proteins found in food provide essential amino acids (EAA) required for the growth, development, and maintenance of various functions in the human body [1]. The nutritional value of a protein food depends on various factors such as amino acid profile, digestibility, and bioavailability, which in turn are dependent on the source (e.g., plant- or animal-based). For example, in plant-based sources, the cell wall rigidity and the presence of other macro/micronutrients and antinutritional components could hinder their applications in food. The food composition and processing conditions also influence nutrient accessibility. Animal-based proteins have been associated with better nutritional value owing to a balanced AA composition and a higher proportion of EAA, e.g., leucine and methionine, compared to plant-based proteins, and better digestibility [2]. However, the production of animal-based proteins such as eggs, meat, and milk can be resource-intensive, including high demand for land use in pasture and for growing feed, freshwater depletion, and intensive agriculture, which has been associated with biodiversity loss and contributes significant greenhouse gas emissions. This has led environmentally conscious consumers to favour plant-based proteins [2].

While substitution with plant-based proteins mitigates some of the environmental and nutritional aspects, it presents a challenge as plant-based products often possess distinctive flavours which may make the product unappealing and also have poor techno-functional properties [3]. Generally, proteins with good functional properties such as solubility, emulsion stability, and foaming ability are of interest in food product development as they imply the food produced will have desired, repeatable, and predictable characteristics. By partial substitution of animal-based proteins with plant-based proteins, the objective of minimising environmental impacts and meeting consumers’ product acceptance could be satisfied. Furthermore, depending on the base ingredients, hybridisation can be tailored to specific nutritional needs. However, there is still a challenge remaining with the techno-functional properties of hybrid proteins as plant- and animal-based proteins have poor interactions [4].

To improve the poor interactions of plant- and animal-based proteins, cross-linking by creating covalent bonds between these protein chains has been proposed; this can be achieved via crosslinkers e.g., dicarbonyl compounds, Maillard-induced glycation, or enzymatic reactions [5]. The enzymatic approach is the most common and preferable because it has a lower risk for the formation of toxic byproducts compared to chemical-based approaches [6]. A challenge with enzymatic cross-linking is that substrate compatibility needs to be considered due to the enzyme’s distinct mechanisms and specificity [5]. For example, laccase, a type of copper-containing polyphenol oxidase, utilises biocatalysts and oxidises amino acids such as tyrosine, cysteine, and tryptophan [7]. Meanwhile, transglutaminase (TG) works by transamidation of particular glutamine residues, specifically catalysing the synthesis of γ-glutamyl isopeptide linkages in proteins, and may be more optimised with food protein containing high glutamine and lysine [8,9].

Among all available enzymes in the market, TG is one of the commonly used enzymes that is considered to be generally safe to eat by the Food and Drug Administration (FDA), although it has been banned in European Union (EU) countries over its potential health risks [10]. Following regulations, the application of laccase may facilitate the treatment required for plant-protein products in the EU region [11]. To the best of the authors’ knowledge, and following a literature search, the application of laccase in cross-linking of plant- and animal-based proteins has not been explored. For example, Jiang et al. [12] only applied laccase to cross-link α-lactalbumin, and Sakai et al. [13] successfully used it to cross-link soy proteins and pectin in plant-based meat analogues. The most recent study in plant- and animal-based protein cross-linking was conducted by Kim et al. [14], in which only TG was used to enhance the emulsifying and encapsulation properties of pea protein isolates using whey protein isolates.

Our study aimed to evaluate the effects of enzymatic cross-linking on the physicochemical and functional properties, including cross-linking pattern, colour, charge distribution (ζ-potential), particle size, thermal stability, morphology, solubility, foaming and emulsifying properties, and total amino acid content of lupin flour (LF) and whey protein concentrate (WPC) mixtures using different enzymes, e.g., two laccases (laccase R, derived from *Rhus vernicifera* and laccase T, derived from *Trametes versicolor*) and TG. Lupin and WPC were chosen due to their increasing popularity in Australia and New Zealand. Two laccases were chosen due to their acceptance as green and sustainable enzymes [15], and TG is an enzyme that has been used widely in many food products and hence a good comparison for laccase performance [8].

## 2. Materials and Methods

### 2.1. Materials

WPC was obtained from Davis Trading Co. Ltd. (Auckland, New Zealand), and LF (*Lupinus angustifolius*) was obtained from Nothing Naughty (Tirau, New Zealand). Precision plus protein™ dual colour standards and Criterion™ TGX™ precast sodium dodecyl-sulfate polyacrylamide gel electrophoresis (SDS-PAGE) gels were purchased from Bio-Rad (Hercules, CA, USA). AccQ.Tag reagent were purchased from Apollo Scientific (Manchester, UK). Other chemicals, including the amino acid standard (A9906, with the addition of asparagine and glutamine), acetic acid, aluminium sulphate, bromophenol blue, colloidal Coomassie Blue G-250, citric acid, dithiothreitol (DTT), ethanol, ferulic acid, glycine, phosphoric acid, sodium dodecyl sulphate (SDS), tri-sodium citrate, tris hydrochloride (Tris-HCl), and urea, were purchased from Sigma Aldrich (Darmstadt, Germany). The enzymes used in this experiment were commercial TG (100 U/g), ACTIVA obtained from Ajinomoto Co. Inc. (Tokyo, Japan), and two different laccases: (1) *Rhus vernicifera* (Laccase R or LR) (50 U/mg) and (2) *Trametes versicolor* (Laccase T or LT) (0.9 U/mg), both purchased from Sigma Aldrich (Darmstadt, Germany).

### 2.2. Proximate Analysis of Lupin Flour and Whey Protein Concentrate Powder

The proximate analysis of LF and WPC powder, including moisture, ash, protein, and fat contents, was determined by standardised methods 934.01, 942.05, 2001.11, and 2003.05, as noted in the AOAC [16]. Carbohydrates were determined by the difference method, which subtracts the sum of the percentages of moisture, protein, fat, and ash from 100.

### 2.3. Enzyme Treatment of Lupin and Whey Protein

LF and WPC were reconstituted at a 4% concentration with Milli-Q water at pH 7.0. For the enzyme treatment, LF and WPC solutions were mixed at a ratio of 1:1 of their protein content (the LF and WPC mixture was labelled as LW). The samples were then preheated at 80 °C for 10 min and cooled to room temperature before the addition of the enzymes. Laccase R and T were added to achieve enzyme activities of approximately 52 and 54 U/g protein, respectively, while TG was added to create an enzyme activity of approximately 13 U/g of protein. TG was added at a lower concentration, as previous testing indicated that the lower concentration still facilitated the protein cross-linking reaction. Laccase was then supplemented with a 25 mM concentration of ferulic acid to influence the enzyme effect, as noted by Sato et al. [17]. The samples were cross-linked for 20 h at 40 °C. For the control, Milli-Q water was added in place of the enzyme. Immediately after treatments, samples were collected for SDS-PAGE analysis and freeze-dried for further analysis.

### 2.4. SDS-PAGE

Samples were analysed by reducing one-dimensional SDS-PAGE using a modified method described by Le et al. [18]. Samples were mixed with Laemmli sample buffer (containing 62.5 mM Tris, 2% SDS, 25% glycerol, and 0.01% bromophenol blue) in a 1:4 (sample:buffer) ratio and reduced with 1M DTT. After heating for 4 min at 95 °C, 20 µL of the sample (containing 4 mg mL^−1^ of protein) was loaded on a precast SDS-PAGE gel (Criterion™ TGX™, Bio-Rad, Hercules, CA, USA). Electrophoresis was subsequently conducted at 200 V for 45 min, using 25 mM Tris, 192 mM glycine, and 0.1% SDS as the running buffer. Following electrophoresis, the gel was stained with colloidal Coomassie Blue G-250 and then destained using 1% acetic acid. The molecular weight (M_w_) of the samples was identified using an M_w_ marker (Bio-Rad, Hercules, CA, USA), covering a range of 10–250 kDa. Gel images were captured using the GelDoc Go imaging system (Bio-Rad, Hercules, CA, USA). Notably, density measurements were not obtained due to the main focus being on cross-linked proteins, and their bands were not well-separated. 

### 2.5. Physicochemical Properties

#### 2.5.1. Colour

The colour of the powder was measured with ColorFlex colorimeter (HunterLab, Reston, VA, USA) to record the CIELAB colour parameters L*, a*, and b*. The powder was spread evenly on a Petri dish. Then, the colour difference (ΔE) was calculated using Equation (1):

(1) $$∆E=\sqrt{{{(L}_{0}-L*)}^{2}{{(a}_{0}-a*)}^{2}{{(b}_{0}-b*)}^{2}}$$

where L_0_, a_0_, and b_0_ are the colour parameter values of the control (LW-C) and L*, a*, and b* are the colour parameter values of the samples treated with enzymes.

#### 2.5.2. ζ-Potential

The ζ-potential values of the protein dispersion were measured using an electrophoretic light scattering instrument (Zetasizer Nano ZS series, Malvern Panalytical Ltd., Malvern, UK). The powders were dispersed into Milli-Q water at a concentration of 0.25% (*w*/*w*) under magnetic stirring at 1000 rpm for at least 15 h. The samples were then diluted 1:1000 in Milli-Q water and centrifuged at 2000 rpm for 10 min to separate large undissolved particles, which allows the avoidance of multiple scattering effects in the measurement. The upper layer of the centrifuged samples was carefully taken and loaded in a measuring cell (DTS1070, Malvern Panalytical Ltd., Malvern, UK). Three readings of ζ-potential values for each measurement were obtained. Three measurements for each sample were performed.

#### 2.5.3. Particle Size Distribution

The particle size and its distribution of protein dispersions were determined by using a laser light scattering analyser (Mastersizer Hydro 3000 SM, Malvern Instruments Ltd., Malvern, UK) connected with a Hydro EV dispersion accessory (Malvern Instruments Ltd., Malvern, UK). The protein dispersions in Milli-Q water at a concentration of 1.0% (*w*/*w*), which was stirred at 1000 rpm for at least 24 h, were used for the measurement. Milli-Q water was used as a dispersant with a refractive index of 1.33. The refractive index and absorption of protein were set at 1.45 and 0.01, respectively. At least two measurements (with four readings for each measurement) for each sample were performed. The results were presented as volume-based mean particle diameter (D[4,3]), surface area-based particle diameter (D[3,2]), and size distribution curves.

#### 2.5.4. Differential Scanning Calorimetry

Protein denaturation of the samples was determined by following the method reported by Anandharamakrishnan et al. [19] using differential scanning calorimetry (DSC823e, Mettler Toledo AG, Greifensee, Switzerland). The samples were dispersed into Milli-Q water at a 15% solid concentration (*w*/*w*), then vortexed at 1000 rpm for 1 min. The vortexed samples were equilibrated at room temperature for at least 30 min with intermittent vortexing. About 15–30 mg of the mixture was pipetted into pre-weighed 40 µL aluminium crucibles (ME-51119870, Mettler Toledo AG, Greifensee, Switzerland) and sealed hermetically with lids. During measurement, a sealed empty crucible was used as a reference, and dry nitrogen at a flow rate of 50 mL min^−1^ was used to purge the measuring cell and prevent moisture condensation. The samples were scanned at a heating rate of 2 °C/min from 20 to 98 °C. The onset, peak and endpoint temperatures, and enthalpy (∆H) of protein denaturation were calculated from the endothermic transition peaks using STARe software version 16.0 (Mettler Toledo AG, Greifensee, Switzerland).

#### 2.5.5. Morphology

The morphology of the powders was analysed using a field emission scanning electron microscope (FESEM, S-4800, Hitachi, Tokyo, Japan). The powder particles were gently fixed on a double carbon tape (which was pre-attached to the metallic stubs), which was then flushed with dry N_2_ gas to remove any unfixed particles. The fixed samples were coated with gold/palladium using a high-resolution sputter coater (208HR, Cressington Scientific Instruments, Watford, UK). The thickness of the coating layer was set at 4 nm. During coating, the samples were steadily rotated to achieve the required thickness homogeneity. The coating step was accomplished with 2 cycles. The coated samples were imaged under FESEM with an accelerating voltage of 10 kV, an emission current of 5 μA, a working distance of 10 mm, and a magnification of 1000. For each sample, at least 5 different positions were imaged.

### 2.6. Functional Properties

#### 2.6.1. Protein Solubility

The solubility of LF, WPC or LW powder was calculated based on the total protein content of the supernatant and the initial sample, as described in Equation (2):

(2) $$Solubility\left(\%\right)=\frac{{TP}_{sup}}{{TP}_{sol}}\times 100$$

where TP_sup_ and TP_sol_ are the total protein content (g) of the supernatant and the total protein content of the LF, WPC or LW solution, respectively.

(i) The supernatant was prepared and collected using the method adapted from Boye et al. [20] and Havea [21] with modifications. LF, WPC or LW powdered samples were redissolved in Milli-Q water to make a 1% (*w*/*v*) concentration, stirred using a magnetic stirrer (200 rpm) for 30 min at 20 °C, and then centrifuged (900× *g*, Gyrozen 1580R, Gyrozen, Gimpo, Republic of Korea) for 10 min at 20 °C.
(ii) To extract all proteins from LF, WPC or LW powder for total protein measurement, the sample was firstly suspended in a cocktail containing 8 M urea, 0.1 M Tris-HCl, and 2% DTT, making up a 1% (*w*/*v*) concentration [22]. The cocktail solution was then agitated in an orbital shaker (600 rpm, ISLD04HDG, Ohaus, Parsippany-Troy Hills, NJ, USA) at 20 °C for 45 min, followed by centrifugation (1000× *g*) for 15 min at 10 °C.

Both the supernatant in (i) and extracted proteins in (ii) were diluted tenfold in the cocktail before applying the Bradford assay [23] for total protein measurements.

#### 2.6.2. Emulsion and Foaming Properties

##### Sample Preparation

The methods used to determine emulsion and foaming properties were adapted from Ho et al. [24] with modifications. Both emulsion and foaming properties followed the same sample preparation. LF, WPC, or LW powders were dissolved in Milli-Q water to make a 1% concentration (*w*/*v*) and stirred using a magnetic stirrer (200 rpm) for 30 min at 20 °C. The solution was equilibrated overnight (20 h) at 4 °C. The samples were then allowed to reach room temperature before 4 mL of each sample was transferred to 15 mL centrifuge tubes for emulsion or foaming testing.

##### Emulsion Ability and Stability

To determine emulsion ability (EA), 2 mL of soybean oil was added into the solution and homogenised using an ultrasonic homogeniser equipped with a Φ10 probe (2BEM-150A, Bueno-Biotech, Nanjing, China). The device was set at 4 kHz with 80% amplitude for 45 s. The pulsing sequence was set to 2.0 s pulse-on and 4.0 s pulse-off. Then, the emulsion was centrifuged (1100× *g*) for 5 min at 20 °C.

The EA was calculated using Equation (3):

(3) $$EA\left(\%\right)=\frac{Height\;of\;the\;emulsified\;layer\;in\;the\;tube\;(mm)}{Height\;of\;the\;total\;content\;in\;the\;tube\;(mm)}\times 100$$

For emulsion stability (ES), the emulsion was heated for 30 min at 80 °C before centrifugation (1100× *g*) for 5 min at 20 °C. The emulsion stability (ES) was calculated using Equation (4):

(4) $$ES\left(\%\right)=\frac{Height\;of\;the\;emulsified\;layer\;after\;heating\;and\;centrifugation\;\left(mm\right)}{Height\;of\;the\;emulsified\;layer\;before\;heating\;\left(mm\right)}\times 100$$

##### Foaming Ability and Stability

To determine foaming ability, the solution was mixed using a homogeniser (25,000 rpm, T-25 digital ULTRA-TURRAX, IKA, Selangor, Malaysia) for 60 s at 20 °C. Foaming ability (FA) was determined by the percentage increase in the volume of protein solution immediately after mixing. The FA was calculated using Equation (5):

(5) $$FA\left(\%\right)=\frac{Height\;of\;the\;foam\;layer\;in\;the\;tube\;(mm)}{Height\;of\;the\;total\;content\;in\;the\;tube\;before\;foaming\;(mm)}\times 100$$

For foaming stability (FS), the foam volume that remained or stabilised after 30 min was recorded and calculated using Equation (6):

(6) $$FS\left(\%\right)=\frac{Height\;of\;the\;foam\;layer\;in\;the\;tube\;after\;30\;min\;(mm)}{Height\;of\;the\;foam\;layer\;in\;the\;tube\;\left(mm\right)\;before\;stabilising}\times 100$$

### 2.7. Amino Acid Measurement

Approximately 25 mg of samples were suspended in 5 mL of 6 M HCl with boiling chips. Samples were then purged with nitrogen for 30 s and hydrolysed at 110 °C for 20 h. After hydrolysis, samples were cooled down to room temperature and filtered using No. 1 Whatman paper. Then, 10 µL of the hydrolysate was taken and suspended in 500 µL of 0.2 M sodium citrate buffer (pH 3), followed by derivatisation using the AccQ.Tag reagent, as previously described by Salazar et al. [25] with some modifications. The derivatised solution was injected into an LC-MS, which consists of a 1260 Infinity Liquid Chromatography coupled with a 6420 Triple Quadrupole Mass Spectrometer (Agilent Technologies, Santa Clara, CA, USA). The chromatographic separation was performed using an Agilent Poroshell EC C-18 column (2.1 × 150 mm, 2.7 µm, Agilent Technologies, Santa Clara, CA, USA). The calibration curve ranged from 0.78–200 µM of amino acid standard mix solution spiked with glutamine and asparagine. In this method, cysteine, methionine, and tryptophan content may be lower than expected due to degradation via acid hydrolysis. Amino acid contents were expressed as g per 100 g protein.

### 2.8. Statistical Analysis

All statistical analyses were calculated using R (version 4.3.1). One-way analysis of variance (ANOVA) was used to evaluate differences between parameters. Tukey’s multiple range test with the criteria *p* < 0.05 was used to determine if there were any significant differences between the parameters. The difference in letters shows the grouping of samples representing significant differences (*p* < 0.05). All results are presented as the mean of triplicates and presented with standard deviation unless stated otherwise.

## 3. Results and Discussion

### 3.1. Proximate Composition of Lupin Flour and Whey Protein Concentrate Powder

The proximate composition of LF and WPC, including moisture, ash, fat, protein, and carbohydrate, is presented in Table 1. The comparison between the composition of LF and WPC used in the study assists in explaining the difference in functional properties of the powders as well as in formulation for the enzyme treatment reaction. Both powders had similar ash and moisture content. Significant differences in the proximate composition were mainly for the protein, fat, and carbohydrate content, in which the last two components present significantly higher content in LF than in WPC, 5.72 vs. 0.81% and 43.26 vs. 23.83%, respectively. Notably, protein content in WPC was 20% higher than in LF. To achieve the desirable 1:1 protein content ratio formulation for the enzyme treatment reaction, more LF was used in the mixture. Consequently, other components, i.e., fat and carbohydrate content, are higher in the mixture compared to the original WPC. These are factors that may alter the functional properties of the mixture [26,27]. The carbohydrates derived from LF could affect the powder’s functional properties as they are composed largely of fibre and may reduce the protein solubility of the mixture. The pH of LF and WPC was also similar, 5.72 and 6.16, respectively. The slight difference in pH could also have an impact on the powders’ functional properties.

### 3.2. SDS-PAGE

**Figure 1:**
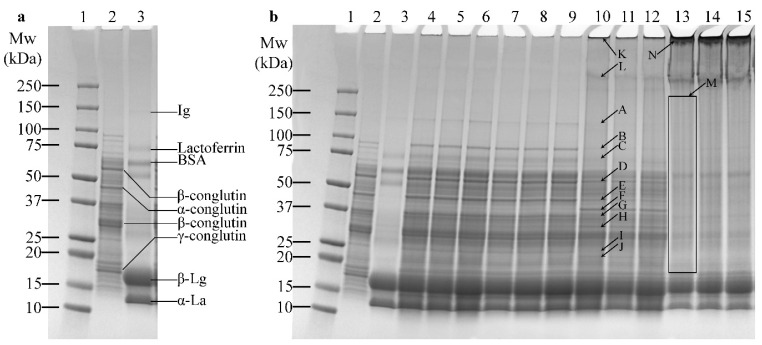
SDS-PAGE of proteins in lupin flour (LF), whey protein concentrate (WPC), non-enzyme-treated LF and WPC (LW-C), and enzyme-treated LF and WPC by laccases (LW-LR and LW-LT) and transglutaminase (LW-TG). (**a**) SDS-PAGE of LF and WPC proteins. Lane 1: protein standard; Lane 2: LF; Lane 3: WPC. (**b**) SDS-PAGE of LF and WPC protein mixtures without and with enzymatic treatment. Lane 1: protein standard; Lane 2: LF; Lane 3: WPC; Lanes 4–6: LW-C (replicates); Lanes 7–9: LW-LR (replicates); Lanes 10–12: LW-LT (replicates); Lanes 13–15: LW-TG (replicates). The big letters (A–N) note the appearance or disappearance of protein bands in the gel.

The protein profile for LF is typically composed of α-, β-, δ-, and γ-conglutin. In a reducing SDS-PAGE, these proteins are presented as monomers such as α-conglutin (~46 kDa), β-conglutin (~65 kDa and ~32 kDa), and γ-conglutin fraction (~18 kDa) (Figure 1a—Lane 2), which are consistent with those previously published by Devkota et al. (2023) [28]. The small fragments of δ-conglutin were reported at ~12 kDa and ~10 kDa in Devkota et al. (2023) [28]; however, these fragments were not found in this LF. This is probably due to the difference in cultivar or less degradation from the processing treatment of the LF. Meanwhile, all the major proteins of WPC can be seen in Figure 1a—Lane 3, including immunoglobulin (Ig) (~160 kDa), lactoferrin (~80 kDa), BSA (~66 kDa), β-lactoglobulin (~18 kDa), and α-lactalbumin (~14 kDa) [29].

Comparing the protein profiles of LF and WPC (Figure 1b—Lanes 2–3) with LW samples (Figure 1b—Lanes 4–15) clearly shows the impact of enzyme treatment on the proteins of LF and WPC. There are additional light bands (~160 kDa or above) and increased darkness of bands (~100 kDa) in LW-C, confirming some interactions of proteins or effects of heating in LF and WPC without enzyme presence (Figure 1b—Lanes 2–3 compared to Lanes 4–6). Regarding the activity of laccase R in protein cross-linking, the bands observed in LW-LR (Figure 1b—Lanes 7–9) were similar to those observed in LW-C (Figure 1b—Lanes 4–6), indicating no visible protein cross-linking. Meanwhile, it was shown that cross-linking occurred in samples LW-LT and LW-TG (Figure 1b—Lanes 10–12 and Lanes 13–15, respectively) according to the disappearance of old bands and the appearance of new bands (mainly high M_w_) in the enzyme-treated samples. In the protein profile of LW-LT, bands from around 160 kDa (A)—equivalent to M_w_ of Ig, 100 kDa (B), 80 kDa (C)—equivalent to M_w_ of lactoferrin, 65 kDa (D)—equivalent to M_w_ of β-conglutin, 46 kDa (E)—equivalent to M_w_ of α-conglutin, 37 kDa (G), and 25 kDa (I), appeared fainter. Conversely, increased density of bands around the size of 40 kDa (F), 30 kDa (H), and 22 kDa (J), as well as persistent protein in the wells (K) and a faint band above the 250 kDa ladder (L), can be observed in Figure 1b—Lane 10. The observation of laccase T’s cross-linking ability aligns with the observation recorded by Sato et al. [17]. Similarly, in the LW-TG protein profile, most proteins between 20 kDa and 250 kDa disappeared (as designated in box M in Figure 1b—Lane 13), and a large dark area in the stacking proteins surrounding the well (N) indicates proteins in monomeric and dimeric forms possibly cross-linked, resulting in large polymers (M_w_ > 250 kDa) accumulated on the top of the gel.

As discussed above, the dark bands observed around the wells of LW-LT and LW-TG protein profiles suggest that large M_w_ proteins were formed during the cross-linking reaction. However, bands in the LW-TG profile were darker, indicating more cross-linking reaction by TG compared to laccase T. This observation could be due to the type and mechanism of the enzymes. TG catalyses lysine and glutamine residues to promote the formation of isopeptide bonds γ-carboxamide groups [8]. Both LF and WPC are rich in glutamine and lysine (discussed later in Section 3.5), making them a suitable substrate for TG-catalysed cross-linking reactions. In the laccase cross-linking reaction between LF and WPC, the oxidative reaction occurs in cysteine, tryptophan, and tyrosine. Both LF and WPC have a low proportion of these amino acids (notably, tryptophan was not detected); therefore, although it may be possible to utilise laccase to cross-link proteins in LF and WPC, the cross-linking reaction may not be as extensive compared to using TG. Overall, in this study, TG performed the best, followed by LT and LR in cross-linking activities.

### 3.3. Physicochemical Properties

#### 3.3.1. Colour

In this study, yellowness was observed in all samples due to the yellow colour of LF and light-yellow colour of WPC. Similarly to its visual appearance, the recorded b* (yellowness) in LF (Table 2) was the highest value amongst the powders’ (b* = 31.76), owing to LF’s natural pigment present in the seed. Meanwhile, WPC’s b* value (b* = 12.49) indicates the powder is still yellow but significantly whiter. The yellowness present in WPC is due to the fat content. Consequently, LW powders were significantly less yellow compared to LF but still more yellow compared to WPC, represented by b* = 21.33, 19.85, 20.95, and 24.50 for LW-C, LW-LR, LW-LT, and LW-TG, respectively. Comparing the yellowness between enzyme-treated and -untreated LW powders, no significant difference was observed in the colours of LW samples treated with both laccases; however, the difference in b* value (yellowness) between LW-C and LW-TG was significant.

The redness was also observed in LW-LT, where the produced powder was visually brown, expressed as a positive a* value of 0.76. The brown colour was unique to LW-LT, as all other samples were visually light yellow and had negative a* values, indicating more greenness in the LAB scale. The browning was attributed to the synthesis of melanin pigments catalysed by the laccase oxidation reaction made from *T. versicolor* [30].

The overall colour difference expressed by ∆E indicated the difference between LW-C and enzyme-treated samples. There were no significant overall colour differences in sample LW-LR and LW-TG with LW-C (∆E = 0.61 and 0.52, respectively), concurring with their visual appearance. Due to the browning of sample LW-LT, the overall colour difference was significant compared to LW-C (∆E = 9.03).

Colour is an important aspect of the physicochemical properties as the colour of food affects consumer preference. For instance, a yellow colour is appealing when incorporated into cereal dishes like pasta and noodles [31], while for milk-like beverages, consumers would prefer a white colour [32], and a brown colour has been associated with a stronger flavour or bitterness [33]. Considering this fact, the colour changes of the LW powders, especially LW-LT with the brown colour, may limit their application in beverage applications.

#### 3.3.2. ζ-Potential

**Figure 2:**
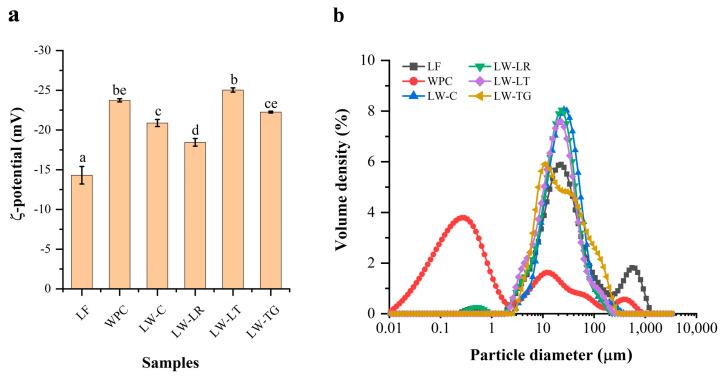
ζ-potential values (**a**) and particle size distribution (**b**) of lupin flour (LF), whey protein concentrate (WPC), non-enzyme-treated LF and WPC (LW-C), and enzyme-treated LF and WPC by laccases (LW-LR and LW-LT) and transglutaminase (LW-TG). For ζ-potential, the values with different letters (a–e) are significantly different among samples (*p* < 0.05).

Cross-linking of proteins can alter their surface charges by affecting their conformation, charged group distribution, and complex formation. ζ-potential is an indicator of the surface charge property of proteins, enabling an investigation into the feasibility of protein cross-linking. As illustrated in Figure 2a, the absolute ζ-potential of LF was 14 mV, significantly lower than that of WPC (24 mV). Proteins with higher ζ-potential tend to demonstrate increased solubility due to enhanced interaction with dipolar water molecules [34]. Therefore, ζ-potential values partially explain the lower solubility of LF compared to WPC (discussed later in Section 3.4.1). Comparison of ζ-potential values between LW-C and enzyme-treated samples (LW-LR, LW-LT, and LW-TG) highlights varying degrees of protein cross-linking in the latter group. These findings align with SDS-PAGE results, where LW-LT and LW-TG exhibited more protein cross-linking, correlating with an increase in absolute ζ-potential values compared to LW-C and LW-LR. Notably, the most pronounced cross-linking occurred in LW-TG according to the visualisation of high M_w_ bands or remnants in the wells of the SDS-PAGE gels. However, LW-LT has the highest ζ-potential values among all cross-linked samples. This could indicate that a certain degree of cross-linking (as in LW-LT) increased the level of surface charge, hence increased ζ-potential values, but a substantial number of cross-linking proteins could aggregate, resulting in a decrease of ζ-potential values, as exemplified by LW-TG. Moreover, the protein surface charge is determined by the balance between hydrophilic and hydrophobic groups [35]. The results suggest that cross-linking with LR could promote the exposure of more hydrophobic groups, leading to a reduced absolute ζ-potential, while cross-linking with TG and LT could expose more hydrophilic groups, resulting in an increased absolute ζ-potential. This observation implies different cross-linking pathways resulting in modification of the protein surface among the investigated enzymes. Variation of ζ-potential values as evidence of protein cross-linking was also reported for soy protein isolate, whey protein isolate [36], and chickpea proteins [37].

#### 3.3.3. Particle Size Distribution

An increase in the particle size of proteins has been reported as an indicator of cross-linking of proteins [36,37]. Here, we report the size of particles and their distribution in LF, WPC and LW samples determined by Mastersizer, and the results are shown in Table 3 and Figure 2b. LF had larger particles than WPC (*p* < 0.05). The size distribution of LF shows a small peak at 0.02–0.7 µm and a large peak at 2–200 µm (Figure 2b), which was similar to the result reported by Bader et al. [38] and Vogelsang-O’Dwyer et al. [39]. In contrast, WPC has a large peak at 0.01–2 µm, along with two smaller peaks at 2–110 µm and 200–800 µm. Mixing LF and WPC, regardless of enzyme treatment, shifts the distribution curves, resulting in a similar curve with a single peak at 2–100 µm observed for all LW samples. Additionally, it increased the surface area-based diameter (D[3,2]). D[3,2] shows higher sensitivity to smaller droplet distributions, while D[4,3] is more responsive to larger droplet distributions [40]. Cross-linked powders, compared to LW-C, did not show a change in D[4,3] but resulted in a decrease in D[3,2]. Overall, the particle size variation was independent of the enzymes used. The results indicated the presence of large and poorly dispersible particles in LW samples. Therefore, high-pressure homogenisation [38] may be necessary to reduce the size and enhance dispersibility for applications in foods, such as beverages or ice creams.

#### 3.3.4. Thermal Properties

The denaturation temperature provides valuable information about the stability of the protein’s structure. Proteins have specific temperature ranges within which they maintain their native and functional conformation. Determining the denaturation temperature allows the determination of the conditions under which a protein is stable or susceptible to unfolding, which is crucial for its applications. Figure S1 illustrates an endothermic peak on the DSC curves of LF, WPC, and LW samples, representing protein denaturation. Table 3 displays the onset, peak, and endpoint temperatures, along with the associated energy (∆H) for this thermal event. The results suggest that LF is more resistant to thermal denaturation than WPC because the thermal change of LF occurred at higher temperatures. WPC exhibited denaturation temperatures ranging from 60–82 °C, consistent with findings reported by Anandharamakrishnan et al. [19] (peak temperature at ~75 °C). Meanwhile, LF showed higher temperatures at 81–88 °C. Our study reports higher denaturation temperatures for LF than those reported by Fontanari et al. [41] (66–70 °C), but lower than Xu and Mohamed [42] (150 °C), and in line with findings from Kiosseoglou et al. [43] (74–95 °C). Discrepancies in reported LF denaturation temperatures could be attributed to variations in LF sources and DSC scanning conditions. The broader denaturation range and higher protein content in WPC (Table 1) resulted in an enthalpy of denaturation five times greater than that of LF.

The results demonstrate that the denaturation temperatures of LF and WPC mixtures (without enzymes) fell within the range of denaturation temperatures observed for LF and WPC individually. Comparing denaturation temperatures between LW-C and enzyme-treated LW samples (LW-LR, LW-LT, and LW-TG) suggests that cross-linking by LT and TG enhances the thermal stability of proteins, particularly the onset temperature. This aligns with similar findings reported for potato proteins cross-linked by TG, laccase, tyrosinase, or peroxidase [44].

#### 3.3.5. Morphology

**Figure 3:**
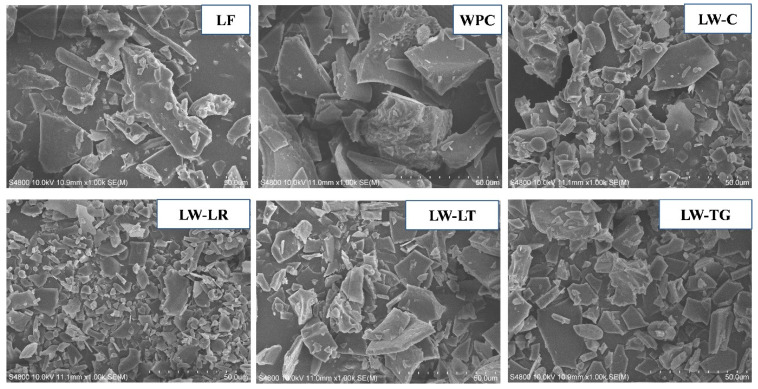
SEM images of lupin flour (LF), whey protein concentrate (WPC), non-enzyme-treated LF and WPC (LW-C), and enzyme-treated LF and WPC by laccases (LW-LR and LW-LT) and transglutaminase (LW-TG). Scale bar = 50 µm.

As indicated in Figure 3, all protein powders exhibited a similar morphology with a broken lamellar structure and as flakes. This morphology is typical of freeze-dried powders [45,46]. The results suggest that the mixing of LF and WPC, particularly with their cross-linking, facilitates the formation of a porous structure in the powder. This structure is prone to breakage during grinding after freeze-drying, resulting in smaller flakes in LW samples compared to LF and WPC. An increase in the porous structure of freeze-dried chickpea proteins due to cross-linking by transglutaminase was also reported [37].

### 3.4. Functional Properties

#### 3.4.1. Protein Solubility

The summarised result of protein solubility can be found in Table 4. WPC presented the highest solubility (98.2%) compared to LF (59%). Considering the composition of LF (Table 1), with equal parts of protein and carbohydrates (mainly fibre), there may be some interference caused by the interaction between the carbohydrates and protein, leading to lower protein solubility of LF. Consequently, LW samples also displayed lower solubility compared to WPC.

The enzyme-treated samples demonstrated a significant impact on protein solubility. LW-LR exhibited higher protein solubility at 91% compared to LW-C at 78.5%. Meanwhile, samples treated with LW-LT and LW-TG exhibited lower protein solubility than LW-C, at 75.2% and 73.7%, respectively. The decrease in protein solubility of plant proteins treated with TG has been previously reported by Nivala et al. [47] and Schlangen et al. [48]. This pattern was explained earlier due to surface charge differences caused by the varying degrees of cross-linking. The observation is interesting as LW-LR, where cross-linking was not visible (Figure 1b, Lanes 7–9), demonstrated improvement in solubility, emulsion, and foaming properties (discussed further in Section 3.4.2). It might be possible that laccase R improves the functional properties of proteins through a different mechanism rather than cross-linking.

When cross-linking occurs, porous structures are formed, leading to a less dense structure that breaks into smaller particles and, in turn, a higher surface charge. As a result, cross-linked powders with more pores have better contact with water, thereby improving protein solubility [49]. However, the opposite occurs with extensively cross-linked proteins. Extensive cross-linking results in a denser structure; hence, it is less porous and has larger powder particles. As evidence, sample LW-LR had smaller powder particles (Figure 3) and a higher solubility as opposed to sample LW-LT and LW-TG, which displayed extensive cross-linking (Figure 1b, Lanes 10–15) and had larger powder particle size (Figure 3). However, the obtained solubility value did not align with the recorded surface charge values, as LW-LT and LW-TG with higher surface charges should have better solubility. Here, we can theorise that the fibres in LF interfered with the surface charge. Isolation of the protein may be required to minimise fibre interference with the surface charge to increase the solubility of LW samples.

#### 3.4.2. Emulsion and Foaming Properties

##### Emulsion Properties

As shown in Table 4, the emulsion ability of the control sample (LW-C) was higher compared to enzyme-treated LW samples (LW-LR, LW-LT, and LW-TG), at 30.3% and 24.24–27.27%, respectively. In contrast, cross-linking treatments improved emulsion stability, with LW-C at 82.13%, and enzyme-treated LW ranging from 89.17–100%. These observations can be attributed to changes in covalent bonds and/or hydrophobic interactions formed during the mixing and cross-linking treatment, leading to a change in protein surface charge and protein aggregation. Grasberger et al. [50] noted a similar observation where the reduction of disulphide bridges occurred in a protein mixture containing whey protein isolate with lupin protein isolate. In later work, the authors also mentioned that the presence of aggregated protein causes bridging flocculation between emulsion droplets, leading to a decrease in emulsion ability [51]. Thus, we could hypothesise that the cross-linking reaction formed high M_w_ proteins and possibly modified the protein structures, leading to a reduction in emulsifying ability. On the contrary, the change in surface charges could enhance the adsorption of the proteins with the oil-water interface, creating the emulsion stability layer and thereby preventing phase separation or improving the emulsion stability [52]. However, the protein interactions in the presence of enzyme could be complex, resulting in an unclear mechanism behind the emulsion ability and stability process of LW mixtures.

Considering the mechanism of the cross-linking and its effect on emulsion properties, LW-LR had the best overall treatment with a minimised decrease in emulsion ability while maintaining a near perfectly stable emulsion. With optimisation of the cross-linking conditions, these properties could potentially be further improved.

##### Foaming Properties

Amongst all samples tested, WPC had the highest foaming ability (169.6%, Table 4), as opposed to LF, with the lowest among the samples (65%). This observation was attributed to the high carbohydrate content noted in Table 1, as the presence of carbohydrates and fat can impact the powder’s functional properties [53]. Lqari et al. [54] reported a higher foaming ability of lupin protein isolate (119%) as compared to LF in this study, which suggests that the use of purified lupin protein may be better than using LF. The addition of WPC to LF without enzyme treatment (LW-C) had a higher increase in foaming ability (126.67%) compared to LF, which was still lower than WPC. A study conducted by Wouters et al. [55] reported a similar observation where partially replacing egg white protein (animal-based protein) with gluten hydrolysate (plant-based protein) resulted in a significant increase in foaming ability in a mixed protein system, regardless of the proportion of protein replaced. Nonetheless, the laccase-treated samples had increased foaming ability to 142.5% and 140% (LW-LR and LW-LT, respectively), while the TG-treated sample resulted in a decrease in foaming ability (120.83%). This decrease could be due to the extensive cross-linking of the powder [56] and could be improved with optimisation.

The foaming stability of LW-C significantly decreased compared to LF, which demonstrates a negative effect of mixing LF and WPC. Alves et al. [57] reported a similar observation in a mixture of soy protein and whey protein isolates. The authors concluded that the air-water interface was disrupted by the presence of less compact plant protein aggregates. When treated with laccase R and TG, the foaming stability of LW samples had slight changes. However, this is not the case with cross-linking reactions with laccase T. The cross-linked structure of protein is very complex, and it is difficult to fully understand the phenomenon. We theorise that the oxidation reaction by laccase T might initiate protein oxidation (as indicated by changes in colour), possibly creating more hydrophilic surfaces (notably LW-LT possesses highest absolute ζ-potential—Figure 2a) which destabilise the foam system, resulting in low foaming stability. Future studies require additional chemical analyses for protein oxidation to elucidate the protein cross-linking pathways in the LF and WPC mixtures.

### 3.5. Amino Acid Content

The amino acid compositions (Table 5) provided an insight into the effects of the cross-linking process on LW’s protein sequences. The mixing of the lupin and whey proteins resulted in an average amount comprising the amino acid content from the two sources. For instance, cystine content, which was 0.26% in LF and 0.86% in WPC, was averaged to 0.62% in LW-C. This trend suggests that the deficiency of certain amino acids from a single protein source can be compensated for by mixing with another source. In terms of nutritional value, WPC contains a higher total EAA content compared to LF, 46.88% vs. 33.41%, respectively. Both values exceed the minimum daily requirement of EAA set by FAO/WHO at 27.7% [58], indicating that both ingredients are excellent protein sources. Nonetheless, the result highlights the importance of hybrid plant- and animal-based protein in the formulation and the synergy between ingredients to tailor the EAA composition based on specific needs.

In the actual values of the amino acid composition across LW samples, there was no significant difference between the EAA profile of LW-C and those of LW-LR and LW-TG. This suggests that the cross-linking treatment (pre-heat treatment and addition of enzyme) had a limited effect on the protein chemical structure. On the contrary, LW-LT presented a slightly reduced amino acid content. The observation can be explained by laccase T’s cross-linking oxidation mechanism, which may have oxidised proteins, allowing interaction with the fats and carbohydrates in LF and WPC to create inactive derivatives [59]; as a result, the modified amino acids generated from the oxidised proteins cannot be detected.

## 4. Conclusions

This study provides insight into strategies for improving the functional properties of plant- and animal-based protein systems through enzymatic cross-linking reactions with laccase and TG. The functional properties of LW mixtures were affected by enzymatic treatment and were dependent on the type of enzyme mechanism and condition used during treatment. Compared with the control (LW with no enzyme), laccase R improved protein solubility, emulsion stability, and foaming ability, while TG improved emulsion stability. Overall, laccases enhanced most of the functional properties better than TG. However, as illustrated by the SDS-PAGE gel, prolonged enzymatic treatment, as with TG, resulted in extensive cross-linking, leading to decreased protein solubility, emulsion ability, and foaming ability. With the oxidative cross-linking mechanism of laccase T, there were adverse effects on protein solubility, emulsion ability and foam stability; in addition, browning of the powder and a decrease in EAA content were observed.

This study has only assessed the physicochemical and functional properties of the LW powder under similar enzymatic treatment. Considering the different mechanisms of laccase and TG, it will be essential to determine the optimal cross-linking condition for each enzyme to obtain the best physicochemical and functional properties of the cross-linked protein powders. Furthermore, the interaction between the ingredients used needs to be investigated. For instance, LF in this study was a more complex system, which may have affected the testing. A simpler system involving isolated proteins, such as lupin protein isolates and whey protein isolates, may provide a more accurate representation of the effects of protein interactions due to cross-linking. It is also necessary to ensure that the modification made does not decrease the amount of nutrients available. Further study will be required to investigate the effect of cross-linking conditions for each enzyme to determine the most optimised cross-linking treatment that could improve the functional properties and to investigate the effect of cross-linking treatment using different combinations of different protein sources.

## Figures and Tables

**Table 1:** Proximate composition and pH of lupin flour (LF) and whey protein concentrate (WPC) powders.

Attributes	LF	WPC
Moisture (%)	6.66 ± 0.21	6.51 ± 0.05
Ash (%)	2.56 ± 0.08	3.79 ± 0.02
Fat (%)	5.72 ± 0.06	0.81 ± 0.01
Protein (%)	43.7 ± 0.90	65.06 ± 0.49
Carbohydrate (%)	43.26 ± 0.13	23.83 ± 0.51
pH	5.72 ± 0.01	6.16 ± 0.01

**Table 2:** LAB colour values of lupin flour (LF), whey protein concentrate (WPC), non-enzyme-treated LF and WPC (LW-C), and enzyme-treated LF and WPC by laccases (LW-LR and LW-LT) and transglutaminase (LW-TG).

Sample	Colour			
	L*	a*	b*	Difference ∆E
LF	83.95 ± 0.66 ^cd^	−0.27 ± 0.08 ^b^	31.76 ± 1.23 ^a^	-
WPC	85.51 ± 1.43 ^b^	−0.01 ± 0.37 ^b^	12.49 ± 0.66 ^d^	-
LW-C	87.64 ± 0.40 ^ab^	−1.63 ± 0.28 ^c^	21.33 ± 0.41 ^c^	0 ^a^
LW-LR	88.74 ± 0.32 ^a^	−1.56 ± 0.03 ^c^	19.85 ± 0.72 ^c^	0.61 ± 0.49 ^a^
LW-LT	82.40 ± 0.62 ^d^	0.76 ± 0.28 ^a^	20.95 ± 0.68 ^c^	9.03 ± 6.3 ^b^
LW-TG	86.20 ± 0.87 ^b^	−1.49 ± 0.15 ^c^	24.50 ± 0.25 ^b^	0.52 ± 0.12 ^a^

L* value represents lightness, (0) = dark, (100) = light; a* value represents red/greenness, (+) value = red, (−) value = green; b* value represents yellow/blueness, (+) value = yellow, (−) = blue. The values with different letters (a–d) in each column indicate significant differences in the colour parameter L*, a*, b*, or ∆E between the samples (*p* < 0.05).

**Table 3:** Average particle size and denaturation temperatures of lupin flour (LF), whey protein concentrate (WPC), non-enzyme-treated LF and WPC (LW-C), and enzyme-treated LF and WPC by laccases (LW-LR and LW-LT) and transglutaminase (LW-TG).

Samples	Particle Size		Protein Denaturation			
	D[4,3], µm	D[3,2], µm	Onset, °C	Peak, °C	Endpoint, °C	∆H (J/g)
LF	111.5 ± 9.2 ^a^	11.5 ± 0.2 ^a^	81.5 ± 2.1 ^a^	83.9 ± 0.2 ^a^	88.3 ± 0.3 ^a^	0.15 ± 0.03 ^a^
WPC	42.1 ± 6.1 ^b^	0.2 ± 0.0 ^b^	60.6 ± 1.0 ^b^	73.2 ± 0.1 ^b^	82.0 ± 1.4 ^b^	0.76 ± 0.03 ^b^
LW-C	34.1 ± 0.2 ^b^	18.6 ± 0.1 ^c^	73.7 ± 1.0 ^a^	84.5 ± 0.4 ^a^	90.1 ± 0.3 ^a^	0.17 ± 0.02 ^a^
LW-LR	31.5 ± 6.1 ^b^	12.5 ± 3.7 ^a^	73.4 ± 1.2 ^a^	83.6 ± 1.6 ^a^	90.1 ± 2.0 ^a^	0.23 ± 0.02 ^a^
LW-LT	30.7 ± 4.2 ^b^	13.5 ± 0.2 ^ac^	77.7 ± 1.6 ^c^	84.7 ± 1.3 ^a^	91.5 ± 2.3 ^a^	0.23 ± 0.08 ^a^
LW-TG	42.7 ± 4.7 ^b^	15.9 ± 0.4 ^a^	78.2 ± 1.3 ^c^	85.2 ± 2.8 ^a^	89.5 ± 3.5 ^a^	0.19 ± 0.03 ^a^

The values with different letters (a–c) in each column indicate significant differences in the particle size or protein denaturation onset, peak, endpoint, and enthalpy (∆H) between samples (*p* < 0.05). The onset, peak and endpoint temperatures, and ∆H of protein denaturation were calculated from the endothermic transition peaks on the DSC curves. D[4,3]: volume-based particle diameter; D[3,2]: surface area-based particle diameter.

**Table 4:** The functional properties of lupin flour (LF), whey protein concentrate (WPC), non-enzyme-treated LF and WPC (LW-C), and enzyme-treated LF and WPC by laccases (LW-LR and LW-LT) and transglutaminase (LW-TG).

Sample	Soluble Protein Content (%)	Emulsion Ability (%)	Emulsion Stability (%)	Foaming Ability (%)	Foaming Stability (%)
LF	59.00 ± 5.29 ^d^	26.66 ± 1.05 ^c^	97.97 ± 3.52 ^a^	65.00 ± 0 ^d^	88.50 ± 0 ^a^
WPC	98.20 ± 0.24 ^a^	90.30 ± 1.05 ^a^	99.33 ± 1.15 ^a^	169.60 ± 5.05 ^a^	7.37 ± 0.25 ^d^
LW-C	78.50 ± 5.49 ^bc^	30.30 ± 1.05 ^b^	82.13 ± 5.5 ^b^	126.67 ± 2.89 ^c^	19.73 ± 0.46 ^bc^
LW-LR	91.00 ± 9.75 ^ab^	26.06 ± 1.05 ^c^	97.63 ± 4.1 ^a^	142.50 ± 0 ^b^	18.10 ± 1.04 ^c^
LW-LT	75.20 ± 5.12 ^c^	24.24 ± 1.05 ^c^	100.00 ± 0 ^a^	140.00 ± 2.50 ^b^	8.93 ± 0.15 ^d^
LW-TG	73.70 ± 5.85 ^c^	27.27 ± 1.82 ^bc^	89.17 ± 6.82 ^ab^	120.83 ± 3.82 ^c^	20.63 ± 1.46 ^b^

The values with different letters (a–d) in each column indicate significant differences in a functional property parameter between samples (*p* < 0.05).

**Table 5:** The total amino acid content determined in lupin flour (LF), whey protein concentrate (WPC), non-enzyme-treated LF and WPC (LW-C), and enzyme-treated LF and WPC by laccases (LW-LR and LW-LT) and transglutaminase (LW-TG), reported in g·100 g^−1^ of proteins.

Sample	LF	WPC	LW-C	LW-LR	LW-LT	LW-TG
Alanine	3.50 ± 0.5 ^b^	5.91 ± 0.7 ^a^	5.40 ± 1.1 ^a^	5.00 ± 0.5 ^ab^	4.48 ± 0.3 ^ab^	5.39 ± 0.4 ^ab^
Arginine	8.98 ± 0.9 ^a^	1.63 ± 0.1 ^c^	5.72 ± 1.6 ^b^	4.64 ± 0.4 ^b^	4.26 ± 0.4 ^bc^	6.32 ± 1.8 ^ab^
Aspartic acid ^1^	11.24 ± 2.0 ^a^	12.24 ± 1.1 ^a^	13.13 ± 2.5 ^a^	12.47 ± 1.5 ^a^	10.80 ± 0.8 ^a^	13.21 ± 1.0 ^a^
Cystine	0.26 ± 0.1 ^c^	0.86 ± 0.1 ^a^	0.62± 0.2 ^ab^	0.59 ± 0.1 ^ab^	0.43 ± 0.1 ^bc^	0.58 ± 0.1 ^ab^
Glutamic acid ^2^	22.50 ± 3.3 ^a^	18.80 ± 2.0 ^a^	22.42 ± 3.9 ^a^	22.95 ± 5.2 ^a^	18.70 ± 1.1 ^a^	22.48 ± 2.2 ^a^
Glycine	4.31 ± 0.9 ^a^	1.94 ± 0.2 ^c^	3.43 ± 0.7 ^ab^	3.03 ± 0.2 ^abc^	2.66 ± 0.1 ^bc^	3.23 ± 0.3 ^abc^
Histidine *	4.09 ± 0.9 ^a^	2.55 ± 0.1 ^a^	3.49 ± 0.8 ^a^	3.31 ± 0.8 ^a^	2.65 ± 0.4 ^a^	3.11 ± 0.4 ^a^
Isoleucine *	3.55 ± 0.5 ^a^	5.43 ± 0.4 ^a^	5.03 ± 0.8 ^a^	4.86 ± 0.5 ^ab^	4.19 ± 0.2 ^ab^	5.04 ± 0.5 ^a^
Leucine *	6.63 ± 0.2 ^c^	10.48 ± 0.5 ^a^	8.69 ± 0.6 ^b^	8.83 ± 0.4 ^b^	9.22 ± 0.5 ^ab^	9.70 ± 0.4 ^ab^
Lysine *	7.44 ± 1.2 ^b^	12.14 ± 1.3 ^a^	11.25 ± 1.8 ^a^	10.39 ± 0.8 ^ab^	9.12 ± 0.7 ^ab^	10.94 ± 1.3 ^a^
Methionine *	ND	ND	ND	ND	ND	ND
Phenylalanine *	4.41 ± 0.8 ^a^	3.60 ± 0.4 ^a^	4.73 ± 0.9 ^a^	4.22 ± 0.5 ^a^	3.42 ± 0.2 ^a^	4.04 ± 0.6 ^a^
Proline	4.65 ± 0.8 ^a^	6.78 ± 0.7 ^a^	6.59 ± 1.2 ^a^	6.19 ± 0.8 ^a^	5.31 ± 0.3 ^a^	6.43 ± 0.6 ^a^
Serine	5.57 ± 0.9 ^a^	5.55 ± 0.5 ^a^	6.33 ± 1.3 ^a^	5.74 ± 0.6 ^a^	4.96 ± 0.3 ^a^	6.01 ± 0.7 ^a^
Threonine *	3.87 ± 0.6 ^c^	7.57 ± 0.8 ^a^	6.69 ± 1.3 ^ab^	6.24 ± 0.6 ^ab^	5.41 ± 0.4 ^bc^	6.52 ± 0.7 ^ab^
Tryptophan *	ND	ND	ND	ND	ND	ND
Tyrosine	3.50 ± 0.8 ^a^	2.73 ± 0.3 ^a^	3.58 ± 0.6 ^a^	3.29 ± 0.5 ^a^	2.51 ± 0.2 ^a^	3.12 ± 0.4 ^a^
Valine *	3.42 ± 0.5 ^b^	5.11 ± 0.5 ^a^	4.89 ± 0.8 ^a^	4.59 ± 0.4 ^ab^	4.02 ± 0.2 ^ab^	4.74 ± 0.6 ^ab^
Total EAA	33.41	46.88	44.77	42.44	38.03	44.09
Total non-EAA	64.51	56.44	67.22	63.9	54.11	66.77

The values with different letters (a–c) in each row indicate significant differences in the amino acid content between samples (*p* < 0.05). ND—not detected. * Essential amino acid. ^1^ Aspartic acid + asparagine. ^2^ Glutamic acid + glutamine.

## Data Availability

The original contributions presented in the study are included in the article/Supplementary Materials, further inquiries can be directed to the corresponding author.

## Supplementary Materials

The following supporting information can be downloaded at: https://www.mdpi.com/article/10.3390/foods13132090/s1, Figure S1. DSC curves indicating the denaturation of lupin flour (LF), whey protein concentrate (WPC), non-enzyme-treated LF and WPC (LW-C), and enzyme-treated LF and WPC by laccases (LW-LR and LW-LT) and transglutaminase (LW-TG).
