# Correction: Santoso et al. Effects of Laccase and Transglutaminase on the Physicochemical and Functional Properties of Hybrid Lupin and Whey Protein Powder. *Foods* 2024, *13*, 2090

**DOI:** 10.3390/foods13223679

**Published:** 2024-11-19

**Authors:** Teguh Santoso, Thao M. Ho, Geerththana Vinothsankar, Kirsi Jouppila, Tony Chen, Adrian Owens, Masoumeh Pourseyed Lazarjani, Mustafa M. Farouk, Michelle L. Colgrave, Don Otter, Rothman Kam, Thao T. Le

**Affiliations:** 1 AUT Centre for Future Foods, Auckland University of Technology, Auckland 1010, New Zealand; 2 School of Science, Auckland University of Technology, Auckland 1010, New Zealand; 3 Department of Food and Nutrition, University of Helsinki, P.O. Box 66, 00014 Helsinki, Finland; 4 Helsinki Institute of Sustainability Science (HELSUS), University of Helsinki, P.O. Box 65, 00014 Helsinki, Finland; 5 Food Technology and Processing, Smart Foods & Bioproducts, AgResearch Ltd., Grasslands Research Centre, Palmerston North 4440, New Zealand; 6 CSIRO Agriculture and Food, 306 Carmody Rd., St. Lucia, QLD 4067, Australia; 7 Australian Research Council Centre of Excellence for Innovations in Peptide and Protein Science, School of Science, Edith Cowan University, Joondalup, WA 6027, Australia; 8 DEO Dairy Consulting, Marton 4787, New Zealand

In the original publication [1], there was a typo on the bottom of Equation (6). A correction has been made as below.

(6) $$FS\left(\%\right)=\frac{Height\;of\;the\;foam\;layer\;in\;the\;tube\;after\;30\;min\;(mm)}{Height\;of\;the\;foam\;layer\;in\;the\;tube\;\left(mm\right)\;before\;stabilising}\times 100$$

Furthermore, there was a mistake in Table 4, where there was a mix-up in the foaming stability values between the control samples. The correct Table 4 appears below. 

**Table 4:** The functional properties of lupin flour (LF), whey protein concentrate (WPC), non-enzyme-treated LF and WPC (LW-C), and enzyme-treated LF and WPC by laccases (LW-LR and LW-LT) and transglutaminase (LW-TG).

Sample	Soluble Protein Content (%)	Emulsion Ability (%)	Emulsion Stability (%)	Foaming Ability (%)	Foaming Stability (%)
LF	59.00 ± 5.29 ^d^	26.66 ± 1.05 ^c^	97.97 ± 3.52 ^a^	65.00 ± 0 ^d^	88.50 ± 0 ^a^
WPC	98.20 ± 0.24 ^a^	90.30 ± 1.05 ^a^	99.33 ± 1.15 ^a^	169.60 ± 5.05 ^a^	7.37 ± 0.25 ^d^
LW-C	78.50 ± 5.49 ^bc^	30.30 ± 1.05 ^b^	82.13 ± 5.5 ^b^	126.67 ± 2.89 ^c^	19.73 ± 0.46 ^bc^
LW-LR	91.00 ± 9.75 ^ab^	26.06 ± 1.05 ^c^	97.63 ± 4.1 ^a^	142.50 ± 0 ^b^	18.10 ± 1.04 ^c^
LW-LT	75.20 ± 5.12 ^c^	24.24 ± 1.05 ^c^	100.00 ± 0 ^a^	140.00 ± 2.50 ^b^	8.93 ± 0.15 ^d^
LW-TG	73.70 ± 5.85 ^c^	27.27 ± 1.82 ^bc^	89.17 ± 6.82 ^ab^	120.83 ± 3.82 ^c^	20.63 ± 1.46 ^b^

The values with different letters (a–d) in each column indicate significant differences in a functional property parameter between samples (*p* < 0.05).

In addition to the change in Table 4, text alterations were made to the Section 3.4.2, second paragraph (fourth sentence) of Foaming Properties:

“When treated with laccase R and TG, the foaming stability of LW samples had slight changes.”

The wording of the Conclusions section, the third sentence in the first paragraph, was also modified to reflect the Corrections to Table 4:

“Compared with the control (LW with no enzyme), laccase R improved protein solubility, emulsion stability, and foaming ability, while TG improved emulsion stability.”

The authors state that the scientific conclusions are unaffected. This correction was approved by the Academic Editor. The original publication has also been updated.

## References

[1] Santoso T., Ho T.M., Vinothsankar G., Jouppila K., Chen T., Owens A., Lazarjani M.P., Farouk M.M., Colgrave M.L., Otter D. (2024). Effects of Laccase and Transglutaminase on the Physicochemical and Functional Properties of Hybrid Lupin and Whey Protein Powder. Foods.

