# Supplementary material for: Effects of Laccase and Transglutaminase on the Physicochemical and Functional Properties of Hybrid Lupin and Whey Protein Powder
Source: Foods. 2024 Jul 1;13(13):2090. doi: 10.3390/foods13132090 (PMC11241515; doi:10.3390/foods13132090)
Supplement: Supplementary file 1 [file foods-13-02090-s001.zip › foods-3041391-supplementary.pdf]

Supplementary Materials

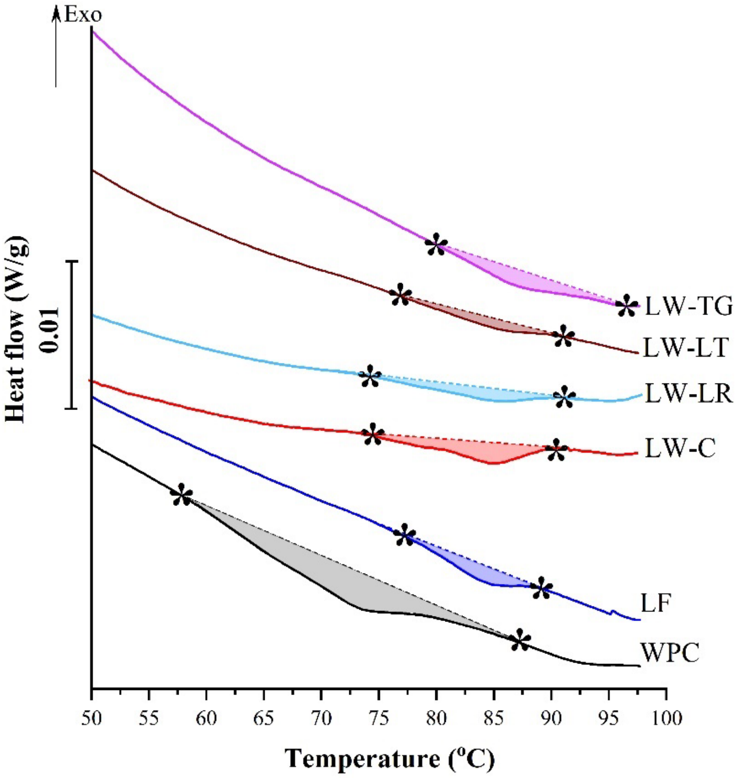

Figure S1. DSC curves indicating the denaturation of lupin flour (LF), whey protein concentrate (WPC), non-enzyme-treated LF and WPC (LW-C), and enzyme-treated LF and WPC by laccases (LW-LR and LW-LT) and transglutaminase (LW-TG).
